# On the Effects of Kernel Configuration in Multi-Kernel Polar Codes †

**DOI:** 10.3390/e24101457

**Published:** 2022-10-12

**Authors:** Souradip Saha, Luis Maßny, Marc Adrat, Peter Jax

**Affiliations:** 1 Fraunhofer Institute for Communication, Information Processing and Ergonomics, Fraunhoferstraße 20, 53343 Wachtberg, Germany; 2 Institute of Communication Systems, RWTH Aachen University, Muffeter Weg 3a, 52074 Aachen, Germany

**Keywords:** polar codes, multi-kernel, 5G

## Abstract

Polar codes are a relatively new family of linear block codes which have garnered a lot of attention from the scientific community, owing to their low-complexity implementation and provably capacity achieving capability. They have been proposed to be used for encoding information on the control channels in 5G wireless networks due to their robustness for short codeword lengths. The basic approach introduced by Arikan can only be used to generate polar codes of length $N=2^{n}$, ∀n∈N. To overcome this limitation, polarization kernels of size larger than 2×2 (like 3×3, 4×4, and so on), have already been proposed in the literature. Additionally, kernels of different sizes can also be combined together to generate multi-kernel polar codes, further improving the flexibility of codeword lengths. These techniques undoubtedly improve the usability of polar codes for various practical implementations. However, with the availability of so many design options and parameters, designing polar codes that are optimally tuned to specific underlying system requirements becomes extremely challenging, since a variation in system parameters can result in a different choice of polarization kernel. This necessitates a structured design technique for optimal polarization circuits. We developed the DTS-parameter to quantify the best rate-matched polar codes. Thereafter, we developed and formalized a recursive technique to design polarization kernels of higher order from component smaller order. A scaled version of the DTS-parameter, namely SDTS-parameter (denoted by the symbol ζ in this article) was used for the analytical assessment of this construction technique and validated for single-kernel polar codes. In this paper, we aim to extend the analysis of the aforementioned SDTS parameter for multi-kernel polar codes and validate their applicability in this domain as well.

## 1. Introduction

Polar codes offer many advantages for channel coding of information bits to be transmitted across a noisy channel, such as low complexity, provably capacity achieving capability and robust characteristics for short codeword lengths. Therefore, they are selected to be used for 5G wireless control channels [1]. Additionally, there are multiple decoding techniques that can be used for the same polar codes, such as Successive Cancellation (SC) [2] and SC-based List [3] or Flip [4] decoding. For a Soft-Input-Soft-Output (SISO) system, Belief Propagation (BP) [5] decoding can also be used. However, on the other hand, certain limitations of polar code design exist as well. This includes the rigid nature of codeword length of the form $N=2^{n}$ due to the originally proposed 2×2 kernel circuit structure in [2]. To address this limitation, well-known rate-matching techniques such as upsizing (by repetition) or downsizing (either by puncturing or shortening) exist. However, this brings in considerable computational overhead, especially if the number of upsized/downsized bits is high. The concept of channel polarization has been generalized to higher polarization kernel sizes, for example, using 3×3 kernel circuits to generate $M=3^{m}$ long codewords as shown in [6,7,8,9]. A combination of multiple kernels of different sizes can be concatenated within the same polarization circuit to design multi-kernel polar codes as provided in [10,11,12,13,14,15,16]. A performance comparison analysis of single kernel polar codes generated by 2×2 and 3×3 kernels and their corresponding multi-kernel counterparts have been performed in [17,18]. It was observed that depending on underlying system requirements, design optimality can vary and in some cases a non-downsized code can outperform a downsized code, therefore being beneficial from both complexity and error rate performance perspectives.

The redundancy of bits in polar codes is implemented by segregating them in two sets, one to encode information bits and the other to encode frozen bits. Ideally, the bits that are polarized to high capacities should be used to encode information bits and the bits with lower capacities for frozen bits. However, different parameters such as channel condition, coderate and codeword length, can create a difference in the optimal choice of the sets of information and frozen bits. A wrong choice could lead to sub-optimal utilization of available channel capacity and consequently sub-optimal error rate performance. By including different kernel circuit designs and multi-kernel layouts in this paradigm, the optimal choice of information and frozen bits is complicated even further. Additionally, for higher kernel sizes, there exist a higher number of possibilities for valid polarization circuits and therefore a higher number of configurations of the corresponding multi-kernel designs. The increment of possible circuit designs to kernel sizes is exponential as shown in [19].

This necessitates a technique to determine the optimal design configuration of polarizing circuits by taking into account all the aforementioned degrees of freedom. It is necessary to determine optimal code design before hardware-based implementation. Additionally, simulating Bit Error Rate (BER) or Block Error Rate (BLER) curves for all possible design configurations is an exceedingly time-consuming process due to an enormous number of design possibilities and the choice of frozen-information bits. Therefore, a simple analytical parameter that can predict polar code design optimality based on system requirements would significantly expand the usability of polar codes for many applications. To address this artifact, ζ parameter was proposed in [19], which in turn was developed from the Downsizing Type-Selection (DTS) parameter from [17]. It was used to quantify the optimality of a specific single kernel design and determine the best choice of kernel circuit over given channel conditions. Since ζ is a scaled version of the DTS parameter, it will be referred to as scaled-DTS or SDTS parameter for the remainder of this paper. The conclusions have been promising since SDTS has proved to be a good estimator of the performance behavior of polarization kernel circuits, validated by corresponding BLER curves. In this paper, we intend to validate the applicability of the SDTS parameter in a multi-kernel polar code circuit design as well.

The rest of the paper is organized as follows. In Section 2, some preliminary information on polarization circuits and multi-kernel designs is provided. The analysis of a multi-kernel configuration using ζ is provided in Section 3. In Section 4, BLER curves are provided to validate the optimality predictions from Section 3. The outlook of the research from the scope of this paper is provided in Section 5. Finally, some concluding remarks are provided in Section 6.

## 2. Preliminaries

### 2.1. 2×2 Kernel Circuit

**Figure 1:**
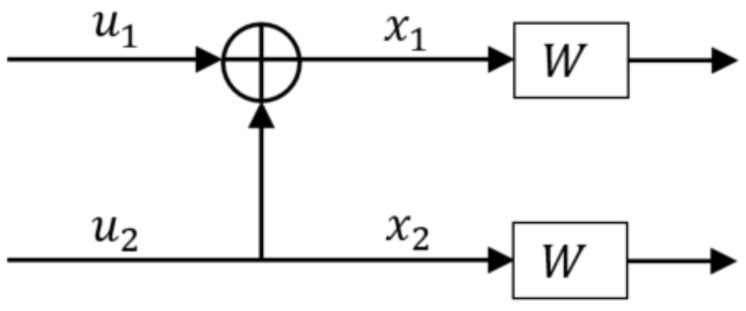
2×2 polarization circuit.

The idea of channel polarization was proposed in [2]. This involves using a 2×2 polarization circuit, as shown in Figure 1, to polarize 2-bit channels of equal capacities to 2-bit channels of unequal capacities. This method of channel polarization can be recursively used to design polarization circuits and thus generate polar codes of length $N=2^{n}$ (for n∈N).

To quantify the channel polarization effect, the *z*-parameter values are obtained as follows [2],

(1) $$\begin{array}{cc} Z_{1} & =2Z\left(W\right)-Z{\left(W\right)}^{2} \end{array}$$

(2) $$\begin{array}{cc} Z_{2} & =Z{\left(W\right)}^{2}\;\;\;\;\;\;\;\;\;\;\;\;\;\;\;\;\; \end{array}$$

where Z(W) denotes the *z*-parameter value of the real channel *W* and $Z_{1}$ and $Z_{2}$ denote the *z*-parameter values of the effective virtual channels. Since, $Z_{1}+Z_{2}=2Z\left(W\right)$, net channel capacity is conserved after polarization. Additionally, as $Z_{1}\geq Z\left(W\right)$ and $Z_{2}\leq Z\left(W\right)$, one virtual channel has higher capacity than the real channel while the other one has lower capacity, creating virtual bit channels with polarized capacities. Although the equality in Equations (1) and (2) is valid just for Binary Erasure Channels (BECs), the concept of channel transformation to polarize their effective capacity holds true for any binary memoryless channel model.

### 2.2. 3×3 Kernel Circuit

**Figure 2:**
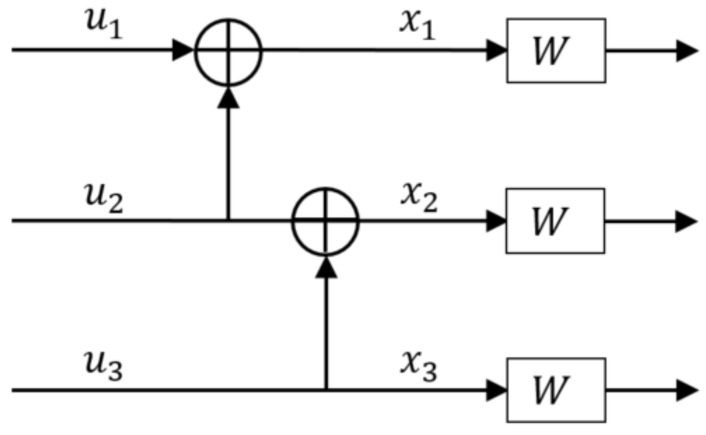
Example of 3×3 polarization circuit [19].

One need not be limited to using just a 2×2 polarization circuit to generate polar codes. The idea of channel polarization can be extended to using any l×l polarization circuit, where l∈N, to generate polar codes of length $l^{n}$. This extends the range of possible codeword lengths with the need for resizing. Using l=3, one can design polar codes of length $N=3^{n}$. An example of a 3×3 polarization circuit is shown in Figure 2, which is same as circuit $C_{3/1d}$ from [19].

The corresponding, *z*-parameter values are as follows,

(3) $$\begin{array}{cc} Z_{1} & =3Z\left(W\right)-3Z{\left(W\right)}^{2}+Z{\left(W\right)}^{3} \end{array}$$

(4) $$\begin{array}{cc} Z_{2} & =2Z{\left(W\right)}^{2}-Z{\left(W\right)}^{3}\;\;\;\;\;\;\;\;\;\;\;\;\;\;\;\;\;\; \end{array}$$

(5) $$\begin{array}{cc} Z_{3} & =Z{\left(W\right)}^{2}\;\;\;\;\;\;\;\;\;\;\;\;\;\;\;\;\;\;\;\;\;\;\;\;\;\;\;\;\;\;\;\;\;\;\;\; \end{array}$$

There have been multiple proposals in the literature to how to polarize 3-bit channels (or how to design the 3×3 polarization circuit) like in the set of articles [6,7,8,9]. In this paper, the focus would be on the 3×3 polarization circuits $C_{3/1d}$ and $C_{3/2x}$ from [19], to analyze the effect of ordering and placement within a multi-kernel circuit layout.

### 2.3. Multi-Kernel Circuit

**Figure 3:**
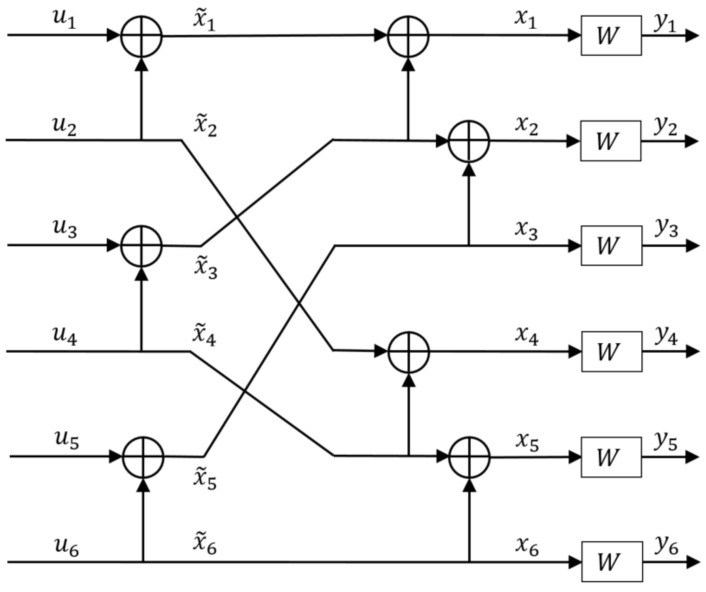
Example of 6×6 polarization circuit using 2×2 and 3×3 polarization circuits [18].

For channel polarization, it is not necessary to limit the recursive usage of a single kernel circuit to polarize bit channels. The polarization effect can also be achieved by using multiple kernel sizes simultaneously within the same encoding/decoding circuits, called multi-kernel polar codes. This extends the scope of codeword lengths even further to increase the versatility. The concept of multi-kernel polar codes is a well-established technique and the corresponding theoretical analysis of channel polarization aspects has been performed in the series of papers [10,11,12,13,14,15,16]. An example of using 2×2 and 3×3 circuits to generate a polar code of length 6 is shown in Figure 3.

The corresponding, *z*-parameter values are given as follows [2],

(6) $$\begin{array}{cc} Z_{1} & =6Z\left(W\right)-15Z{\left(W\right)}^{2}+20Z{\left(W\right)}^{3}-15Z{\left(W\right)}^{4}+6Z{\left(W\right)}^{5}-Z{\left(W\right)}^{6} \end{array}$$

(7) $$\begin{array}{cc} Z_{2} & =8Z{\left(W\right)}^{2}-16Z{\left(W\right)}^{3}+14Z{\left(W\right)}^{4}-6Z{\left(W\right)}^{5}+Z{\left(W\right)}^{6}\;\;\;\;\;\;\;\;\;\;\;\;\;\;\;\;\;\;\; \end{array}$$

(8) $$\begin{array}{cc} Z_{3} & =4Z{\left(W\right)}^{2}-4Z{\left(W\right)}^{3}+Z{\left(W\right)}^{4}\;\;\;\;\;\;\;\;\;\;\;\;\;\;\;\;\;\;\;\;\;\;\;\;\;\;\;\;\;\;\;\;\;\;\;\;\;\;\;\;\;\;\;\;\;\;\;\;\;\;\;\;\;\;\;\;\;\;\;\; \end{array}$$

(9) $$\begin{array}{cc} Z_{4} & =3Z{\left(W\right)}^{2}-3Z{\left(W\right)}^{4}+Z{\left(W\right)}^{6}\;\;\;\;\;\;\;\;\;\;\;\;\;\;\;\;\;\;\;\;\;\;\;\;\;\;\;\;\;\;\;\;\;\;\;\;\;\;\;\;\;\;\;\;\;\;\;\;\;\;\;\;\;\;\;\;\;\;\;\; \end{array}$$

(10) $$\begin{array}{cc} Z_{5} & =2Z{\left(W\right)}^{4}-Z{\left(W\right)}^{6}\;\;\;\;\;\;\;\;\;\;\;\;\;\;\;\;\;\;\;\;\;\;\;\;\;\;\;\;\;\;\;\;\;\;\;\;\;\;\;\;\;\;\;\;\;\;\;\;\;\;\;\;\;\;\;\;\;\;\;\;\;\;\;\;\;\;\;\;\;\;\;\;\;\;\;\;\;\;\; \end{array}$$

(11) $$\begin{array}{cc} Z_{6} & =Z{\left(W\right)}^{4}\;\;\;\;\;\;\;\;\;\;\;\;\;\;\;\;\;\;\;\;\;\;\;\;\;\;\;\;\;\;\;\;\;\;\;\;\;\;\;\;\;\;\;\;\;\;\;\;\;\;\;\;\;\;\;\;\;\;\;\;\;\;\;\;\;\;\;\;\;\;\;\;\;\;\;\;\;\;\;\;\;\;\;\;\;\;\;\;\;\;\;\;\;\;\;\;\;\; \end{array}$$

Using a different kernel size in each stage or a different order of kernel sizes in different stages or even different kernel designs of the same size, one can generate different polarization circuits for the same codeword length $K=l^{n1}\times m^{n2}\times \cdots \times p^{ns}$ for l,m,p,n1,n2,ns∈N. In this article, we focus just on such multi-kernel polarization circuits of the form $K=2^{n1}\times 3^{n2}$ using 3×3 circuits $C_{3/1d}$ and $C_{3/2x}$ from [19]. This would be the baseline for our analysis of kernel placement and ordering in multi-kernel polarization circuits in the subsequent sections.

## 3. Analysis of Kernel Configuration

Multi-kernel polar codes not only provide higher flexibility in the choice of polarization kernel, but they also offer the opportunity to arrange the kernels in any arbitrary order. Due to the non-commutativity of the Kronecker product, different kernel orders yield different polarization circuits and generator matrices, i.e., different polar codes of the same length. The effect of kernel order has not been extensively discussed in the literature and a systematic approach to the optimization of the kernel order is near non-existent. In this paper, we investigate the effect of the kernel order on practical codeword lengths by assessing the polarization effect using the *z*-parameter. Analogously to [19], ζ ($=\sum_{i\in I}Z_{i}$), the upper bound of (7) from [19], is used as BLER estimate.

### 3.1. Effect of Kernel Order on *z* Parameter

Similar to the approach used in [19], in this paper, we would use the SDTS parameter to analyze the polarization behavior and correspondingly determine the optimal circuit configurations for a given set of system parameters. Therefore, the effect of different kernel orders can be studied by *z* and effectively SDTS parameters. The purpose of this section is to formalize the connection between the *z*-parameter behavior for different kernel orders.

Let f(Z) ($f{\left(Z\right)}_{C_{n}}^{\left(i\right)}:\left[0,1\right]\to \left[0,1\right]$) denote the evolution of *z*-parameter values, where *n* denotes kernel size and identification of circuit $C_{n}$ and *i* denotes the bit index. The functional values are exemplified by (1) and (2) for kernel size 2, where $f{\left(Z\right)}_{C_{2}}^{\left(1\right)}\equiv \left(1\right)$ and $f{\left(Z\right)}_{C_{2}}^{\left(2\right)}\equiv \left(2\right)$. Similarly, since Figure 2 corresponds to circuit $C_{3}/1d$, (3)–(5) provide the $f{\left(Z\right)}_{C_{3/1d}}$ values, i.e., $f{\left(Z\right)}_{C_{3/1d}}^{\left(1\right)}\equiv \left(3\right)$, $f{\left(Z\right)}_{C_{3/1d}}^{\left(2\right)}\equiv \left(4\right)$ and $f{\left(Z\right)}_{C_{3/1d}}^{\left(3\right)}\equiv \left(5\right)$. As given in [19], the generator matrices corresponding to polarization circuits $C_{n1}$ and $C_{n2}$, are denoted as $G_{n1}$ and $G_{n2}$. Considering a simple example of the two-stage concatenation of kernel circuits of two different sizes, the resultant generator matrix is,

(12) $$G_{n}=G_{n1}⊗G_{n2}$$

where n=n1·n2. There are n/n1=n2 blocks of $C_{n1}$ circuits in stage 1 and n/n2=n1 blocks of $C_{n2}$ circuits in stage 2. The *j*th bit channel of the *i*th block in stage 1 is connected to the *i*th bit channel of the *j*th kernel in stage 2. This is the structural makeup for multi-kernel polar codes. Thus, the *z*-parameter value of output bit channels of *j*th kernel block from stage 1 to stage 2 is $f{\left(Z\right)}_{C_{n1}}^{\left(j\right)}$. Since the kernel block $C_{n2}$ is repeated n1 times in stage 2, and each of them employ the same recursive *z*-parameter formulae, in stage 2, $f{\left(Z\right)}_{C_{n2}}^{\left(k\right)}$ is applied to the bits of first kernel block (k=1,…,n2), $f{\left(Z\right)}_{C_{n2}}^{\left(k-n2\right)}$ for the second kernel block (k=n2+1,…,2·n2) and so on, until the bit n=n1·n2. Effectively, the *z*-parameter value $Z_{k}$ for the *k*th input bit channel can be recursively computed as,

(13) $$Z_{k}=\left\{\begin{array}{cc} f{\left(Z\right)}_{C_{n2}}^{\left(k\right)}\left(f{\left(Z\right)}_{C_{n1}}^{\left(1\right)}\right) & k=1,\ldots ,n2\\ f{\left(Z\right)}_{C_{n2}}^{\left(k-n2\right)}\left(f{\left(Z\right)}_{C_{n1}}^{\left(2\right)}\right) & k=n2+1,\ldots ,2\cdot n2\\ .\\ .\\ .\\ f{\left(Z\right)}_{C_{n2}}^{\left(k-(n1-1)\cdot n2\right)}\left(f{\left(Z\right)}_{C_{n1}}^{\left(n2\right)}\right) & k=(n1-1)\cdot n2,\ldots ,n1\cdot n2 \end{array}\right.$$

The aforementioned (13) can be generalized as,

(14) $$Z_{k}=f{\left(Z\right)}_{C_{n2}}^{\left(1+((k-1)\;mod\;n2)\right)}\left(f{\left(Z\left(W\right)\right)}_{C_{n1}}^{\left((k-1)\;div\;n2\right)}\right)$$

where *W* denotes the real bit channel. At the final output stage, for simplicity, we denote (14) as $f{\left(Z\right)}_{C_{n2}}^{\left(k2\right)}\circ f{\left(Z\right)}_{C_{n1}}^{\left(k1\right)}$, for kl=1,…,nl. Generalizing it to a concatenation of a arbitrary number kernels $C_{n1},C_{n2},\ldots ,C_{ns}$, the resulting *z*-parameter values at the circuit output is given as $f{\left(Z\right)}_{C_{ns}}^{\left(ks\right)}\circ \cdots \circ f{\left(Z\right)}_{C_{n2}}^{\left(k2\right)}\circ f{\left(Z\right)}_{C_{n1}}^{\left(k1\right)}$. Therefore, the task of optimizing the kernel order can be denoted by the following optimization problem,

(15) $$f{\left(Z\right)}_{C_{n\;\pi_{s}}}^{\left(k\;\pi_{s}\right)}\circ \cdots \circ f{\left(Z\right)}_{C_{n\;\pi_{2}}}^{\left(k\;\pi_{2}\right)}\circ f{\left(Z\right)}_{C_{n\;\pi_{1}}}^{\left(k\;\pi_{1}\right)}\left\{Z\left(W\right)\right\}=\min\zeta$$

where π is used to denote the permutation of ordering the kernel circuits such that kernel $C_{ni}$ is assigned to stage *i*, i.e., $\pi =\{\pi_{1},\pi_{2},\ldots ,\pi_{s}\}$ with $\pi_{1}\in \left\{n1,n2,\ldots ,ns\right\}$, $\pi_{2}\in \left\{n1,n2,\ldots ,ns\right\}\left\{\pi_{1}\right\}$, $\pi_{3}\in \left\{n1,n2,\ldots ,ns\right\}\left\{\pi_{1},\pi_{2}\right\}$ and so on. This gives the optimal kernel order for a given codeword length and the required number of information bits.

Limiting the analysis for two different kernels and sizes, we divide the aforementioned optimization problem into the following degrees of freedom to be tuned:

1. The order in which the sets of two kernels would be concatenated;
2. The position (stage #) at which the second kernel is to be placed if used just once.

Case 1 involves determining whether a kernel is used either in the initial or in the latter stages. Case 2 is used to determine at which intermediate stage would it be optimal to place a different kernel. The goal is to identify regularities that can be generalized to any kernel.

### 3.2. Evolution of SDTS Parameter for Variation of Kernel Order

In this section, we analyze the first case of kernel ordering mentioned in Section 3.1. Here, two different kernels (of different sizes) are concatenated to design the polarization circuit. The different kernels are arranged in separate blocks of stages and are not nested. Since only two kernels are considered, the assessment involves placing a set of kernels on either the encoder input or output side of the polarization circuit. For the rest of this paper, the encoder output would be denoted as the first stage (stages close to the encoder input are initial stages) and the encoder input as the last stage (stages close to the encoder output are latter stages). In this section, we will focus only on kernels of sizes 2 and 3. In [19], four polarization circuits have been developed with different polarization characteristics, amongst which groups of two shared similar behavior with each other. We will use circuits $C_{3/1d}$ and $C_{3/2x}$ for the analysis in this section as they have shown to depict contrary behavior in polarization circuit designs. Simulations over different values of the total number of stages have shown that the polarization exponents (a metric proposed in [20] to quantify the strength of polarization for near infinite codeword length) of either $C_{n1}$ or $C_{n2}$ have no effect on the optimal kernel order. Since we would like to investigate codewords of practical codeword (finite) lengths, the exponent would not be the most reliable indicator of design optimality anyway.

**Figure 4:**
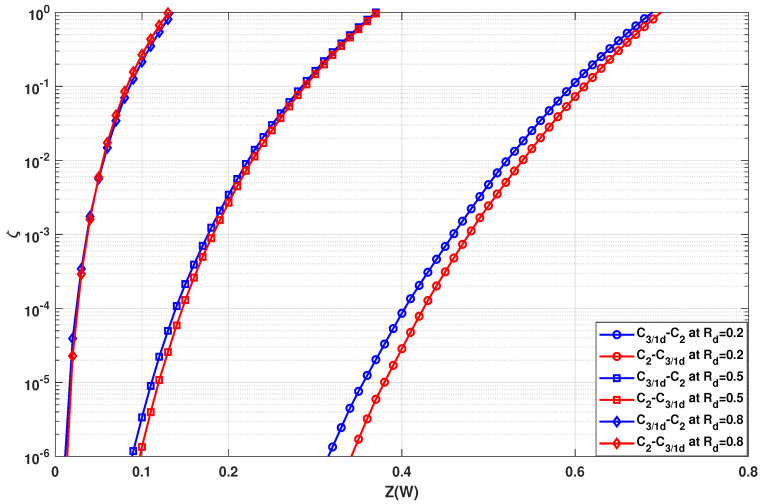
Evolution of SDTS parameter using n1=2 and n2=3 with circuit $C_{3/1d}$ for N=216.

**Figure 5:**
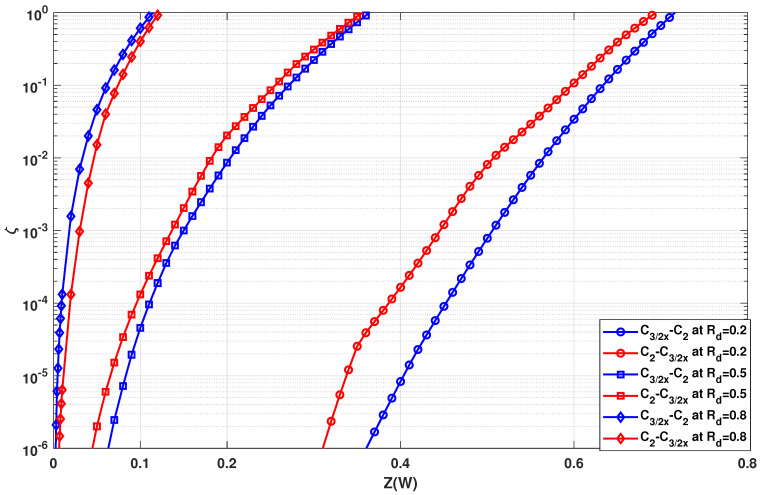
Evolution of SDTS parameter using n1=2 and n2=3 with circuit $C_{3/2x}$ for N=216.

Using n1=2 and n2=3 with three stages each ($N=2^{3}\cdot 3^{3}=216$), the evolution of values of ζ is shown in Figure 4 and Figure 5. In Figure 4, circuit $C_{3/1d}$ is used as the $C_{3}$ circuit whereas in Figure 5 circuit $C_{3/2x}$ is used as the $C_{3}$ circuit. In both figures, the blue curves denote the circuits where $C_{3}$ kernels are used in the initial stages of the polarization circuit. On the other hand, the red curves denote the circuits where $C_{3}$ kernels are used in the latter stages of the polarization circuit. It has been observed that for high values of ζ (typically for high coderate and high value of Z(W)), the curves are too close to each other. Therefore, the range of ζ values can be ignored. On the other hand, the comparison of ζ for very low values would not be accurate since the difference in polarization quantified by ζ would be negligible. Hence, the range of very low ζ values would also be ignored.

In Figure 4, it is observed that $C_{3/1d}$ is more effective when placed at the latter stages. There is a clear gap between the ζ curves of the two kernel order permutations at low coderate ($R_{d}=0.2$) and half coderate ($R_{d}=0.5$) as seen in Figure 4. At high coderate, however, the ζ curves of the different kernel orders are very close to each other. For high coderate, the ζ curves interchange their order, with the blue one being slightly better for high ζ values and the red one being slightly better for low ζ values. Due to the close proximity of ζ curves, the effectiveness of ζ needs to be verified by BLER simulations, as to whether the order is truly irrelevant when using this combination at high coderates.

In Figure 5, it is observed that it is preferable to use kernel $C_{3/2x}$ in the initial stages for low coderates ($R_{d}=0.2$) and half coderate ($R_{d}=0.5$). However, it is advantageous to use this kernel in the latter stages for very high coderates. It was shown in [19] that $C_{3/2x}$ is a good choice just for low coderates. Therefore, the best circuit order involving $C_{3/2x}$ for high coderates is of low practical importance. Although the analysis was performed in a single-kernel paradigm, the evaluation of the SDTS parameter is the same in a multi-kernel paradigm as well.

Comparing Figure 4 to Figure 5, the SDTS parameter performance of multi-kernel polar codes based on kernel $C_{3/1d}$ is worse than that of multi-kernel polar codes based on kernel $C_{3/2x}$ for low coderates ($R_{d}=0.2$ and $R_{d}=0.5$). On the other hand, the SDTS parameter performance of multi-kernel polar codes based on kernel $C_{3/1d}$ is better than that of multi-kernel polar codes based on kernel $C_{3/2x}$ for higher coderates ($R_{d}=0.8$). Hence, when $C_{3/1d}$ is used in multi-kernel circuits, the performance can be optimized by placing them at the latter stages of the polarization circuit. Similarly, when using $C_{3/2x}$, the performance can be optimized by placing them at the initial stages of the polarization circuit.

Overall, a general best kernel order, that fits all design parameters, is difficult to determine, since the behavior of the SDTS parameter depends on the coderate, the particular kernel used and the corresponding kernel order. Nevertheless, based on desired system parameters, optimized circuit design and kernel choices can be determined and vice-versa.

### 3.3. Evolution of SDTS Parameter for Variation of Kernel Position

In this section, we analyze the second case of kernel ordering mentioned in Section 3.1. Here, the effect of different positions of a single kernel inside a multi-kernel polar code is analyzed. To simplify the analysis, we will consider only one stage of the $C_{3}$ kernel and the remaining n−1 stages of the $C_{2}$ kernel, for *n* being the total number of stages in the polarization circuit. The $C_{3}$ kernel is placed in one of the intermediate or the first or last stages. Depicting position number by pos, pos∈[1,n]. The resulting codeword length would be of the form $N=2^{\left(n-1\right)}\cdot 3$. As in Section 3.2, we will only use kernel circuits $C_{3/1d}$ and $C_{3/2x}$ for $C_{3}$ kernel circuits. To further simplify the analysis, in this sub-section ζ would be assessed just for half coderate values ($R_{d}=0.5$).

**Figure 6:**
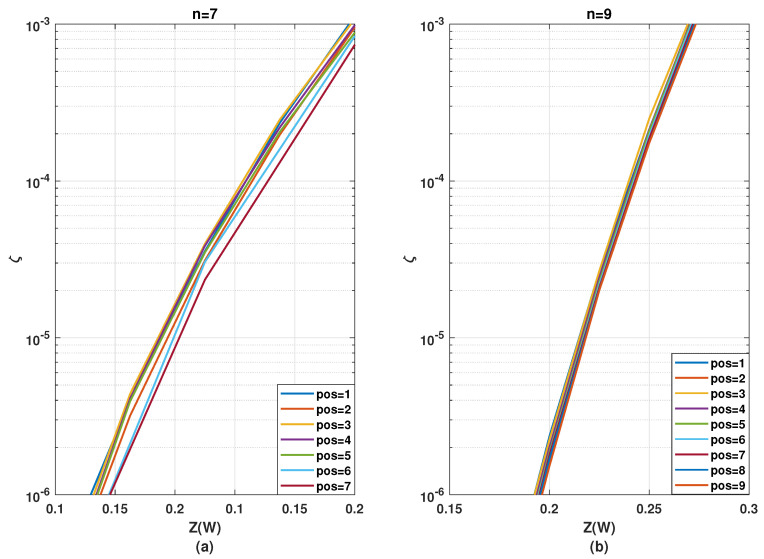
Evolution of SDTS parameter for N=192 and N=768 with n−1=6 stages (**a**) or n−1=8 stages (**b**) of $C_{2}$ circuits and one intermediate stage of $C_{3/1d}$ circuit with pos being its stage number.

Figure 6 depicts the evolution of the SDTS parameter, by placing one kernel $C_{3/1d}$ at different intermediate stages of a polarization circuit composed along with (n−1)={6,8} stages of $C_{2}$, i.e., in total n={7,9} stages or N={192,768}. The position that yields the best ζ performance differs for varying *n*. For the n=7, placing $C_{3/1d}$ at the last stage leads to the best performance. For n=9, the optimal position of $C_{3/1d}$ is barely distinguishable. Therefore, as a generalization, it would be safe to conclude that placing kernel $C_{3/1d}$ at the latter stage yields near-optimal performance. A justification for such behavior is that since $C_{3/1d}$ provides a high number of relatively good bit channels, it is best suited for the last stages, where the bit channels are already polarized to some extent and the overall polarization needs to be improved further. Since the ζ curves are very close, however, the exact performance comparison would require verification with BLER simulations.

**Figure 7:**
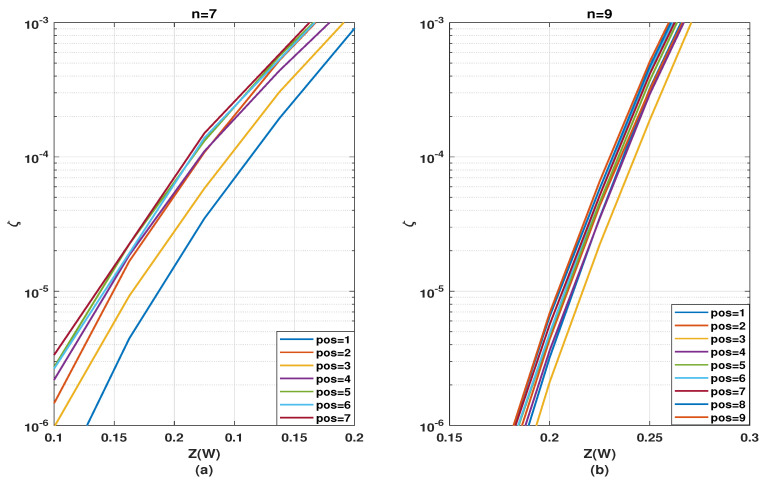
Evolution of SDTS parameter for N=192 and N=768 with n−1=6 stages (**a**) or n−1=8 (**b**) of $C_{2}$ circuits and one intermediate stage of $C_{3/2x}$ circuit with pos being its stage number.

The same analysis is presented for different positions of $C_{3/2x}$ in Figure 7. For the n=7 (N=192), placing $C_{3/2x}$ at the first stage leads to the best performance, whereas, placing $C_{3/2x}$ on the last stage yields the worst performance. For n=9 (N=768), placing $C_{3/2x}$ at the third position yields best performance. Thus, there is a general tendency that $C_{3/2x}$ yields a better ζ performance at the initial rather than at the latter stages. A justification for such behavior is due to the availability of fewer good bit channels in $C_{3/2x}$. This is less effective at the latter stages because the quality of the majority of the already polarized bit channels is not significantly improved. Employing this kernel in the initial stages, however, generates some highly polarized bit channels, if not many. These highly reliable channels can be used in the succeeding stages to further improve the reliability of other channels.

A general observation is that performance optimality based on kernel position gets gradually less relevant with the increasing number of stages. This pattern is as expected since, with an increasing number of stages, the polarization effect from one specific stage gets diluted and becomes less significant within the overall polarization circuit. Therefore, it is more important to optimize the kernel order for a smaller number of stages, i.e., small finite codeword lengths. Additionally, $C_{3/1d}$ yields better performance when placed at the latter stages, whereas $C_{3/2x}$ is more effective at the initial stages. Such behavioral differences of $C_{3/1d}$ and $C_{3/2x}$ with respect to coderates have already been observed in [19]. Hence, the kernel order of a multi-kernel polar code has to be optimized individually for each kernel implementation.

**Figure 8:**
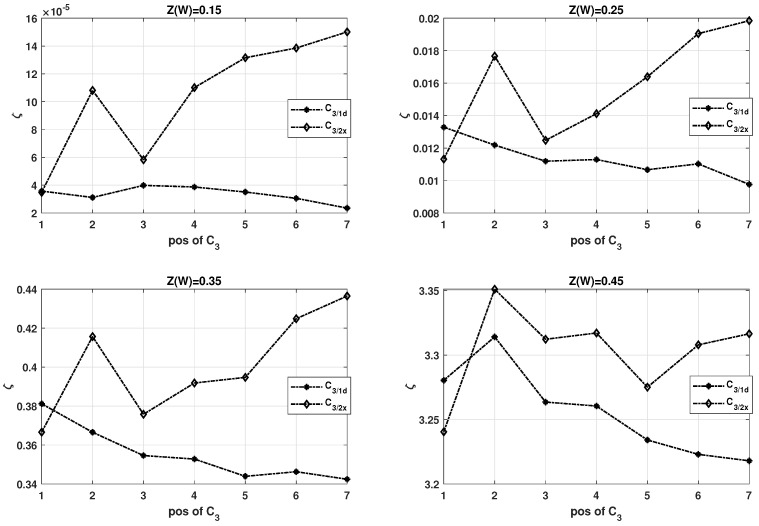
Polarization profile, using SDTS parameter, when $C_{3/1d}$ and $C_{3/2x}$ are placed at first or last stages of a polarization circuit with n=7.

In order to validate this hypothesis, a profile of the polarization over the position of $C_{3}$ kernel at various stages for a seven stage circuit (n=7) and $R_{d}=0.5$ is presented in Figure 8 under different channel conditions (values of Z(W)). This polarization profile shows how ζ varies when $C_{3}$ circuits ($C_{3/1d}$ or $C_{3/2x}$) are placed at one of the intermediate or first or last stages of the circuit.

From each of the four subplots, one can observe that it is beneficial to place $C_{3/2x}$ in the initial stages of the circuit as it results in a smaller ζ value. Eventually, for the latter when placed in the latter stages, $C_{3/1d}$ offers a smaller ζ value, therefore being the better choice of $C_{3}$. This observation holds true for any channel condition (multiple instances of Z(W)). An interesting observation from Figure 8 is that the curve corresponding to $C_{3/1d}$ is predominantly monotonically decreasing by a small factor over subsequent stages. This is because $C_{3/1d}$ can improve the overall polarization in subsequent stages owing to the presence of a high number of relatively good bit channels. On the other hand, the curve corresponding to $C_{3/2x}$ tends to have a contradictory behavior due to fewer reliable bit channels of this kernel. Only when applied at the first stage, the succeeding stages can exploit the few highly reliable bit channels to eventually improve other bit channels.

## 4. Error Rate Performance

In Section 3, we used the SDTS parameter to analyze the optimality of the order and position of different kernel circuits within a multi-kernel polar code configuration. It was observed that there does not exist a universal configuration of optimal polar code design that performs best for any given codeword length or coderate. However, SDTS provides an indicator of the trends in the polarization of a certain circuit design and how it would perform for a given set of network parameter requirements.

Based on Section 3, the best position of a kernel within a multi-kernel polar code depends on the coderate on the one hand, and on the kernel implementation on the other hand. For $C_{3}$ kernels, the preferable position of $C_{3/1d}$ within a multi-kernel polar code is one of the latter stages, while the preferable position of kernel $C_{3/2x}$ is one of the initial stages. These ζ-based predictions would be verified using block error rate (BLER) curves in the following sub-sections. All simulations have been performed over an Additive White Gaussian Noise Channel (AWGNC), Binary Phase Shift Keying (BPSK) is the underlying modulation scheme and SC decoder is the polar decoding technique. The complexity of SC decoding [2] depends only on the codeword length and is independent of the kernel configuration and ordering. In Section 4.1, the observations from Section 3.2 are validated and in Section 4.2 the observations from Section 3.3 are validated.

### 4.1. BLER Performance for Variation of Kernel Order

**Figure 9:**
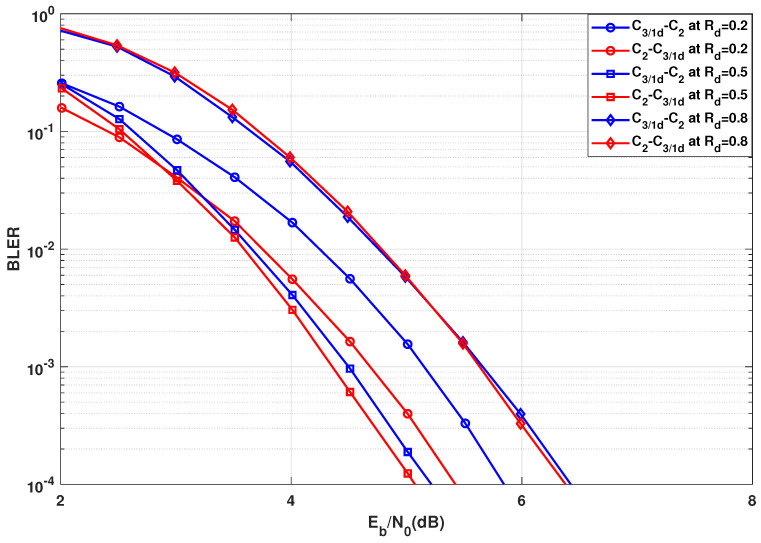
BLER performance comparison using $C_{3/1d}$ in the initial or latter stages for N=216.

**Figure 10:**
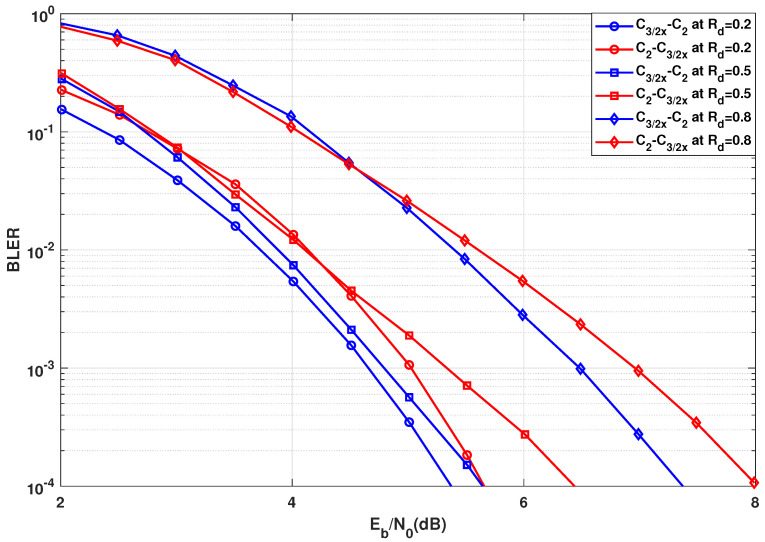
BLER performance comparison using $C_{3/2x}$ in the initial or latter stages for N=216.

In this section, the BLER results are presented for the case in Section 3.2, where the transformation network consists of two groups of different kernels. The error rate performance corresponding to Figure 4 are presented in Figure 9 and the error rate performance corresponding to and Figure 5 are presented in Figure 10. As per the system configuration in Section 3.2, $N=2^{3}\cdot 3^{3}=216$, implying three stages of $C_{2}$ and $C_{3}$ kernels each. The blue BLER curves correspond to the $C_{3}$ kernels being used in the initial stages of the polarization circuit, whereas the red BLER curves correspond to the $C_{3}$ kernels being used in the latter stages of the polarization circuit.

In Figure 9, it is observed that BLER performance is best when kernel $C_{3/1d}$ is used at the latter stages for any given coderate. However, for low coderate, the performance difference is substantially high, approximately 0.2 dB for $R_{d}=0.2$. For half or high coderates, the performance gap is negligible with positioning $C_{3/1d}$ at the latter stages slightly outperforming the positioning at the initial stages. For high coderate ($R_{d}=0.8$) too, the performance gap is almost negligible with a flip in performance curves at low and high BLER ranges. This behavior corresponds exactly to the predicted behavior from Figure 4, hence proving the effectiveness of using the SDTS parameter to predict BLER performance.

In Figure 10, it is observed that the BLER performance is best when kernel $C_{3/2x}$ is used at the initial stages for any given coderate, with approximately 0.7 dB for $R_{d}=0.5$. One exception is for high coderate at high BLER values when placing $C_{3/2x}$ at the latter stages is a better option, which is predicted by Figure 5. However, since low BLER is not of high interest for performance analysis, this observation can be ignored.

Comparing Figure 9 to Figure 10, clearly for low coderate ($R_{d}=0.2$), the BLER performance is better when $C_{3/2x}$ is used over $C_{3/1d}$. On the other hand, the BLER performance is better when $C_{3/1d}$ is used over $C_{3/2x}$ at high coderate ($R_{d}=0.8$). This further validates the prediction of optimality of $C_{3/2x}$ for low coderate and $C_{3/1d}$ for high coderate mentioned previously as well as determined in [19]. Most importantly, the BLER performance concurs with the observations from Section 3.2. The SDTS parameter performance predicted the best kernel order correctly. The only wrong prediction was made for kernel $C_{3/2x}$ for high coderate value ($R_{d}\approx 0.8$) at low BLER. As $C_{3/2x}$ has been deemed unsuitable for high coderates anyway, this inconsistency is not highly relevant for practical use cases, as $C_{3/1d}$ would be the preferred kernel choice for higher coderates.

### 4.2. BLER Performance for Variation of Kernel Position

**Figure 11:**
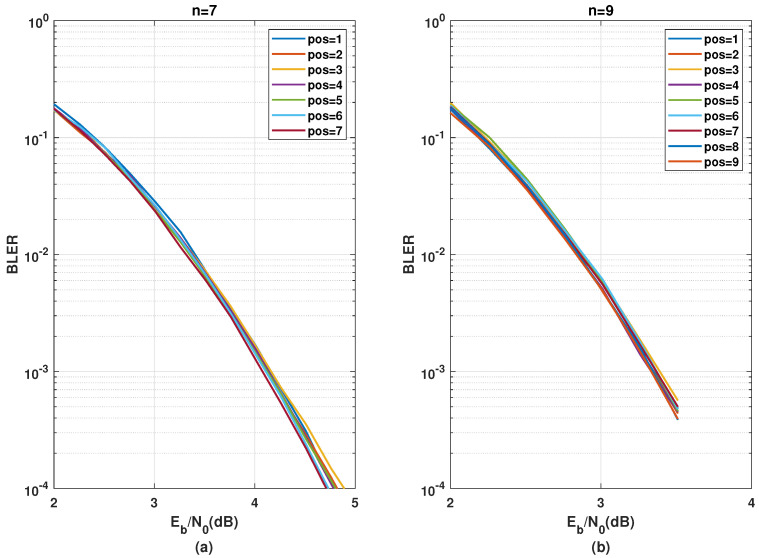
BLER performance comparison using $C_{3/1d}$ in the initial or latter stages for n=7 stages (**a**) and n=9 stages (**b**).

**Figure 12:**
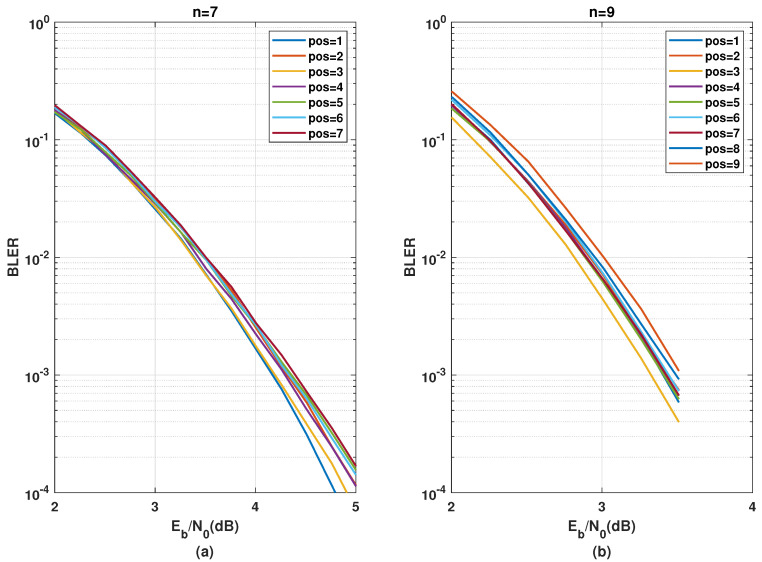
BLER performance comparison using $C_{3/2x}$ in the initial or latter stages for n=7 stages (**a**) and n=9 stages (**b**).

In addition to the group-wise kernel order permutation, the SDTS parameter performance of different positions for a $C_{3}$ kernel in combination with multiple stages of $C_{2}$ kernels was analyzed in Section 3.3. The error rate performance corresponding to Figure 6 are presented in Figure 11 and the error rate performance corresponding to and Figure 7 are presented in Figure 12.

Figure 11 shows that placing the kernel $C_{3/1d}$, at the last stage yields the best BLER performance, while placing it at the third stage provides the worst performance for both n=7 and n=9 polarization circuits. This complies with the predictions from the SDTS parameter in Section 6 that kernel $C_{3/1d}$ is most effective when placed on the last stages. Comparing Figure 11a to Figure 11b, the performance difference is smaller (more negligible) for a higher number of stages, i.e., for n=9 compared to n=7, which corresponds to the observation (Figure 6a compared to Figure 6b) that for a polarization circuit with a higher number of stages, the performance dependency on kernel position for a single stage is substantially diluted.

Figure 12 shows that placing the kernel $C_{3/2x}$, at the first stage for n=7 and at the third stage for n=9 yields the best BLER performance, while placing $C_{3/2x}$ at the last stage provides worst performance. This complies exactly with the observations from Section 5. It is beneficial to apply kernel $C_{3/2x}$ in the initial stages.

The authors of [11] presented a similar approach to kernel positioning. They concluded that the kernel considered therein ($G_{3}$ with polarization exponent the same as $C_{3/1d}$) is preferable in the latter stages for half coderate. As already conjectured above, kernels with the same or a similar exponent may have the same optimal kernel order. Overall, the optimization of kernel order turns out to be a complex problem. One challenge is the high number of parameters that affect the performance of a kernel order, such as the particular kernel design, codeword length and the coderate. Another issue is the lack of ability to generalize the observations since the optimization of kernel order proved to be a problem specific to the considered code design. Nevertheless, the behavioral tendency of a particular kernel implementation could be verified, for example, whether it tends to be advantageous either in the initial or in the latter stages of the polarization circuit. This has been demonstrated for the kernels $C_{3/1d}$ and $C_{3/2x}$.

## 5. Future Work

The effectiveness of the SDTS parameter has been successfully investigated for polarization kernels of sizes 2, 3 and 4 in the single kernel and sizes 2 and 3 in the multi-kernel paradigm. Naturally, an extension of this research would include identifying how useful ζ could be for arbitrarily higher kernel sizes and their multi-kernel implementations. Since, the number of valid kernels increases exponentially with increasing kernel size, there exist many possible candidates and consequently their combinations. Hence, it becomes increasingly complex to determine optimal configurations of polarization circuit designs. The availability of such a parameter to quantify the strength of polarization for finite codeword lengths without error rate simulations is quite useful. Since the SDTS parameter is aimed for the same purpose as the polarization exponent, identifying a relationship between these two parameters could be an interesting future research work that would significantly improve generalized polar code design for any codeword length.

## 6. Conclusions

The performance characteristics of higher ordered kernels have been identified by the SDTS parameter and its utility for optimization of polarization circuits to generate polar codes has been validated for single kernel polar codes in [19] and multi-kernel polar codes in this paper. Polarization kernels of higher order are desirable to improve the applicability of polar codes since a wider range of codeword lengths can be achieved without the need for downsizing techniques such as puncturing or shortening. The SDTS parameter has been able to accurately predict the preferable choice of kernel configuration as well as ordering and placement within the circuit structure to provide the best error rate performance of finite length codewords for a given set of system parameter settings. This offers a much simpler and quicker approach for polar code design without the need for BER/BLER simulations to determine the optimality of polar code design. Although the BLER validation has been performed using only the SC decoder, we performed some baseline simulations using SCL (Successive Cancellation List) decoding with list sizes 2 and 8, well as the corresponding SCL with CRC (Cyclic Redundancy Check) of 8 bits. The observed behavior was the same as for the SC decoder, i.e., the SDTS parameter can make accurate error rate performance prediction over all those SC-based polar decoding techniques.

For the 3×3 kernels investigated, it has been determined that $C_{3/1d}$ is better at the latter and $C_{3/2x}$ is better at the initial stages. Additionally, the performance difference for $C_{3/1d}$ curves is small and for $C_{3/2x}$ is large. This is because $C_{3/2x}$ has few highly polarized channels and therefore a change in their placement within the polarization circuit has a higher effect on the performance. On the other hand, since $C_{3/1d}$ has a higher number of less polarized bit channels, the effect of a change in their placement within the polarization circuit has a lower effect on the performance. All these effects are predicted by the SDTS parameter and thus it could provide insights into kernel behavior within a polarization circuit. This aids in generalizing the optimal design of polarization circuits for a wide range of codeword lengths which is one of the key challenges associated with the implementation of polar codes.

## Data Availability

Data available on request and not publicly accessible due to ownership restrictions.

## Abbreviations

The following abbreviations are used in this manuscript:

AWGNC	Additive White Gaussian Noise Channel
BEC	Binary Erasure Channel
BER	Bit Error Rate
BLER	Block Error Rate
BP	Belief Propagation
BPSK	Binary Phase Shift Keying
DTS	Downsizing Type Selection
SDTS	Scaled-DTS
SISO	Soft Input Soft Output
SC	Successive Cancellation
